# Transcriptome-Wide Cleavage Site Mapping on Cellular mRNAs Reveals Features Underlying Sequence-Specific Cleavage by the Viral Ribonuclease SOX

**DOI:** 10.1371/journal.ppat.1005305

**Published:** 2015-12-08

**Authors:** Marta Maria Gaglia, Chris H. Rycroft, Britt A. Glaunsinger

**Affiliations:** 1 Program in Molecular Microbiology, Sackler School of Graduate Biomedical Sciences, Tufts University, Boston, Massachusetts, United States of America; 2 Department of Molecular Biology and Microbiology, Tufts University School of Medicine, Boston, Massachusetts, United States of America; 3 School of Engineering and Applied Sciences, Harvard University, Cambridge, Massachusetts, United States of America; 4 Department of Mathematics, Lawrence Berkeley National Laboratory, Berkeley, California, United States of America; 5 Howard Hughes Medical Institute, University of California, Berkeley, Berkeley, California, United States of America; 6 Department of Plant and Microbial Biology, University of California, Berkeley, Berkeley, California, United States of America; University of Southern California, UNITED STATES

## Abstract

Many viruses express factors that reduce host gene expression through widespread degradation of cellular mRNA. An example of this class of proteins is the mRNA-targeting endoribonuclease SOX from the gamma-herpesvirus Kaposi’s sarcoma-associated herpesvirus (KSHV). Previous studies indicated that cleavage of messenger RNAs (mRNA) by SOX occurs at specific locations defined by the sequence of the target RNA, which is at odds with the down-regulation of a large portion of cellular transcripts. In this study, we address this paradox by using high-throughput sequencing of cleavage intermediates combined with a custom bioinformatics-based analysis pipeline to identify SOX cleavage sites across the mRNA transcriptome. These data, coupled with targeted mutagenesis, reveal that while cleavage sites are specific and reproducible, they are defined by a degenerate sequence motif containing a small number of conserved residues rather than a strong consensus sequence. This degenerate element is well represented in both human and KSHV mRNA, and its presence correlates with RNA destabilization by SOX. This represents a new endonuclease targeting strategy, in which use of a degenerate targeting element enables RNA cleavage at specific locations without restricting the range of targets. Furthermore, it shows that strong target selectivity can be achieved without a high degree of sequence specificity.

## Author Summary

The human tumor virus Kaposi’s sarcoma-associated herpesvirus uses a messenger RNA (mRNA)-targeting nuclease called SOX to cut cellular RNAs, thereby controlling gene expression in infected cells. How SOX causes destruction of the majority of mRNAs while at the same time achieving target and cut site specificity has remained unknown. To resolve this paradox, we mapped the location of SOX cut sites across the human RNA transcriptome, and confirmed that SOX recognizes specific RNA sequences. These sequences have some conserved features but are otherwise quite distinct. Thus, SOX uses a degenerate motif to achieve broad targeting capability while preserving cut site specificity within the RNA. Little is currently known about the targeting mechanisms of many host and viral ribonucleases, and thus these data provide a framework for understanding how these proteins may operate.

## Introduction

Triggering wide-spread RNA degradation is a common strategy that viruses use to decrease host gene expression, also known as host shutoff [1,2]. Viral factors from many different families including herpesviruses, coronaviruses and orthomyxoviruses either directly cut RNAs or indirectly stimulate RNA cleavages in an endonucleolytic fashion [3,4]. Cellular RNA exonucleases are then recruited to degrade the fragments, resulting in a reduction in RNA and consequently protein levels [3]. Despite the fact that the proposed role of most of these host shutoff ribonucleases (RNases) is to modulate immune responses, they are generally thought to have little or no specificity and to affect host messenger RNAs (mRNAs) indiscriminately. However, increasing evidence suggests that this view may be overly simplistic, and that some of the RNases display selectivity for or against specific targets [5–12]. This type of specificity may provide an additional level of regulation in viral control of the host transcriptome. How this selectivity is achieved and how it is balanced with the widespread shutoff phenotype remain open questions.

The SOX family of proteins from gamma-herpesviruses is an example of a viral RNase that displays both broad targeting of RNAs and a poorly understood level of selectivity. Gamma-herpesviruses include the human pathogens Kaposi’s sarcoma-associated herpesvirus (KSHV), which causes Kaposi’s sarcoma as well as lymphomas in immunocompromised individuals and remains a leading cause of cancer-linked death in sub-Saharan Africa. The SOX (ORF37) protein is expressed early during the lytic cycle of KSHV infection and its expression triggers RNA degradation, which is recapitulated by expression of the protein alone [13]. Homologs of SOX in the other human gamma-herpesvirus, Epstein Barr virus (EBV BGLF5), and in the model murine pathogen murine herpesvirus 68 (MHV68 muSOX) also degrade RNA in cells [14,15]. Studies in MHV68 suggest that host shutoff by the SOX family of proteins is crucial for viral replication in specific cell types and for systemic spread of the virus and establishment of a latent infection [16]. Transcriptomic studies of mRNA levels during KSHV or MHV68 infection and in cells overexpressing SOX demonstrate that this family of proteins triggers the degradation of a majority of both host and viral transcripts [6,7,17]. However, in-depth mechanistic studies of SOX reveal a more complex picture. SOX targets mRNAs, as opposed to non-coding RNA species, a specificity that is related to the association of SOX with polyribosomes [5]. Moreover, selected transcripts, like the cytokine interleukin 6 (IL-6) [6] and apoptosis enhancing nuclease (AEN) [7], are spared from SOX-mediated decay. In the case of IL-6, protection is conferred by the presence of a protective sequence in the 3’ untranslated region (UTR) [8], but AEN appears to be intrinsically resistant to SOX mediated degradation [7], without a clear protective element in its sequence. The most unexpected observation, however, is that KSHV SOX and EBV BGLF5 cut RNAs at specific locations that appear to be determined by an unknown targeting element [3,5]. These specific cleavages become apparent upon knockdown of the human RNase Xrn1, the major 5’-3’–directed RNase in eukaryotic cells, which is responsible for clearing the 3’ RNA fragments generated by these RNases [5]. This ability of SOX to cut at specific locations within mRNA yet target the majority of transcripts argues for a degenerate targeting motif.

In general, the principles guiding the positioning of RNA cleavages by cellular and viral mRNA endonucleases are not well understood. In the case of endonucleases, the term “sequence specificity” is sometimes used to refer to preferential cutting at specific dimers, often inferred from *in vitro* studies (for example in Datta et al. [18]). However, this specificity cannot explain cutting of RNAs at single locations in mRNAs. Additional specificity can be conferred by localization of the target mRNA to a specific site in the cells [19] or proximity to a “landmark” feature on the RNA, such as the 5’ of the transcript [20,21] or the stop codon location [22]. The SOX targeting system is unprecedented because the sequence of the targeting element alone appears to direct cleavages in mRNA, and because the targeting element is longer than 25 nt [5].

To address how SOX specificity is mediated, we applied a degradome sequencing technique called parallel analysis of RNA ends (PARE) [23] to map cleavage sites of the SOX protein across the human transcriptome. Development of a stringent Python-based analysis algorithm, which we term PyDegradome, allowed identification of SOX-dependent cuts at specific locations across the mRNA transcriptome. The sequences surrounding these sites contained no strong consensus sequence, but rather a degenerate sequence pattern that nonetheless conferred specificity when analyzed experimentally in endogenous mRNA targets. The presence of a more complex targeting motif explains how SOX achieves cleavage specificity without sacrificing target breadth, and offers a framework for understanding how additional viral and cellular endonucleases may operate.

## Results

### Development of a novel bioinformatics pipeline to detect high-confidence SOX cleavage sites across the transcriptome following PARE

Prior analyses of individual mRNAs indicated that the KSHV RNase SOX cuts at specific locations within the RNA, in a manner dependent on the sequence surrounding the cleavage site [5]. By performing 5’ rapid amplification of cDNA ends (5’ RACE) on the GFP reporter mRNA, we found that the GFP mRNA was cleaved in the same location regardless of whether SOX was transiently expressed in 293T cells or expressed from the KSHV genome in lytically-reactivated iSLK.219 cells (S1 Fig). This is in agreement with the fact that SOX activity does not rely on any additional viral proteins, and that its cleavage activity can be studied in transfected cells [5,7,13].

**Fig 1:**
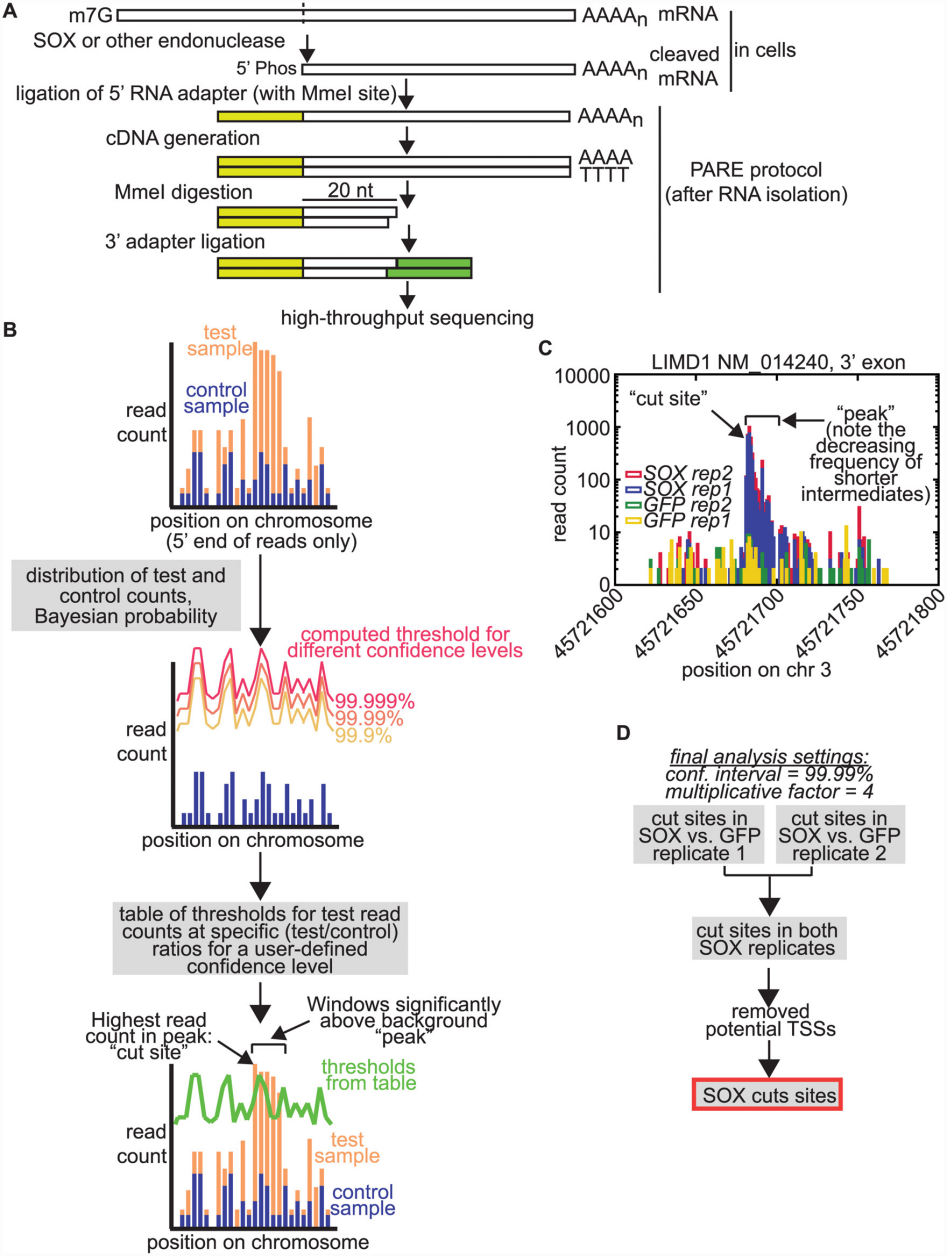
PARE experimental procedure and peak finding analysis pipeline. A) Diagram of the PARE procedure. B) Schematic of PyDegradome analysis approach, which uses read counts in a control sample to generate a table of thresholds to compare test sample counts to. The table lists thresholds for a particular user-defined confidence level and for a range of ratios between control and test samples. The applicable ratio for each position is computed by multiplying a user-defined multiplicative factor by the ratio of total read counts for the exon in test vs. control samples, thus accounting for variation in RNA levels and total mapped reads. Read counts in test sample at each position must exceed the threshold to be identified as part of a peak. C) Example of plot of read counts (5’ end only) for 200 nt surrounding a SOX cut site identified by PyDegradome within the 3’–most exon of the LIMD1 NM_014240 transcript in the four samples. Note that y-axis has a logarithmic scale. This example shows the expected distribution for a cut site followed by exonucleolytic degradation due to residual Xrn1 activity or to the action of another nuclease, with the highest read count at cut site and decaying counts at following positions. Positions referred to as “peak” and “cut site” in the text are marked on the graph. D) Flow chart of steps used to defined SOX cut sites used for further analyses.

To dissect the specificity of SOX cleavage across the mRNA transcriptome, we designed an approach to map SOX cleavage sites in endogenous mRNAs using PARE [23,24]. PARE is an RNA-seq based methodology that allows mapping of the 5’ ends of uncapped, phosphorylated RNA species, such as mRNA degradation fragments (Fig 1A). As we previously found that the SOX degradation intermediates are cleared by the host 5’-3’ exonuclease Xrn1 [5], we stabilized degradation intermediates in 293T cells expressing a GFP-SOX fusion [7] by knocking down Xrn1 (S2A Fig). Cells expressing GFP alone were used as controls to filter out RNA fragments generated by cellular RNases or other processing events. This was important because multiple studies have shown that PARE and similar techniques detect a large number of RNA fragments in human cells, many of which are of unknown origin [25–27]. We prepared and sequenced PARE libraries from two replicates of SOX-expressing or GFP control cells and extracted the 5’ end of each mapped read, which represents the cleavage site (S1 Table).

Conventional analysis of PARE and similar degradome datasets relies on detecting cut sites in each condition and then comparing conditions to each other *a posteriori*, but initial attempts at detecting and validating cut sites indicated that this approach did not provide sufficient discriminatory power to identify SOX-specific cleavage sites. In previous studies using PARE or similar approaches [26,27], additional information about the pathways, such as the miRNA sequences for miRNA cut site studies or the proximity of the site to a stop codon for studies of SMG6 and nonsense mediated decay, was used to further select “true” cut sites. However, such contextual information does not exist for SOX cleavage specificity. Therefore, we devised a Python-based analysis approach that would directly use our control dataset as a baseline, which we termed PyDegradome (Fig 1B). The analysis uses a Bayesian probability framework to determine whether the read counts at a given location differ significantly between control and test samples, taking into account random variations in the number of reads. Using Bayes' theorem, we determine for each location whether the underlying rate of fragment production in the test sample is a multiplicative factor larger than the control rate at a user-defined confidence level. The user also chooses the multiplicative factor. For a given control read count, we thus compute a threshold that the read counts in the test samples have to exceed to be considered part of a “peak” (Fig 1B). To improve efficiency when testing thousands of locations, the software builds a reference table of the thresholds for the entire dataset. This approach allowed the identification of locations within the transcriptome where the read counts were statistically higher in the samples from SOX-expressing cells than in control samples (peaks). In order to correct for up- or down-regulation of the RNAs and for the total number of reads obtained for each sample, the ratio used to determine thresholds was computed from the user-defined multiplicative factor and the ratio of the total number of reads mapping to each exon in test vs. control samples (Fig 1B). To prevent isolated high read counts from skewing our analysis, we integrated read counts within small windows (4 nt) rather than single nucleotides. Adjacent windows that passed the cutoff were then combined into a single peak. We optimized the user-defined confidence level and ratio by determining how many peaks were detected when comparing each SOX replicate sample to its GFP control to detect SOX-specific peaks, or performing the opposite comparison to detect GFP-specific peaks. In addition, we also ran the program to detect peaks specific to only one biological repeat, by comparing the two SOX or GFP replicates to each other, as these peaks may represent experimental noise. Although we consistently detected more SOX-specific peaks than GFP-specific peaks, varying the parameters improved discrimination of SOX-specific peaks (S2B Fig) and reduced detection of “noise” peaks specific to one repeat. Based on this optimization, we empirically set the final iteration of the program to detect 4 nt windows with read counts in the test samples that are four fold higher than read counts in the control samples within a confidence level of 99.99%. Because these parameters are conservative, we expect that the SOX cut sites we detected do not represent a comprehensive list of all SOX cut sites, but rather only the highest confidence sites. Within each peak, we also determined the position where the read count was highest, and we considered this position the location of putative cut site (with the cleavage occurring 5’ of this position) (Fig 1B and 1C). With similar optimization, this program could be used to identify the ends of degradation fragments in other degradome datasets that contain matching test and control samples.

### SOX cuts sites are abundant and not positioned relative to landmark features of mRNA

**Fig 2:**
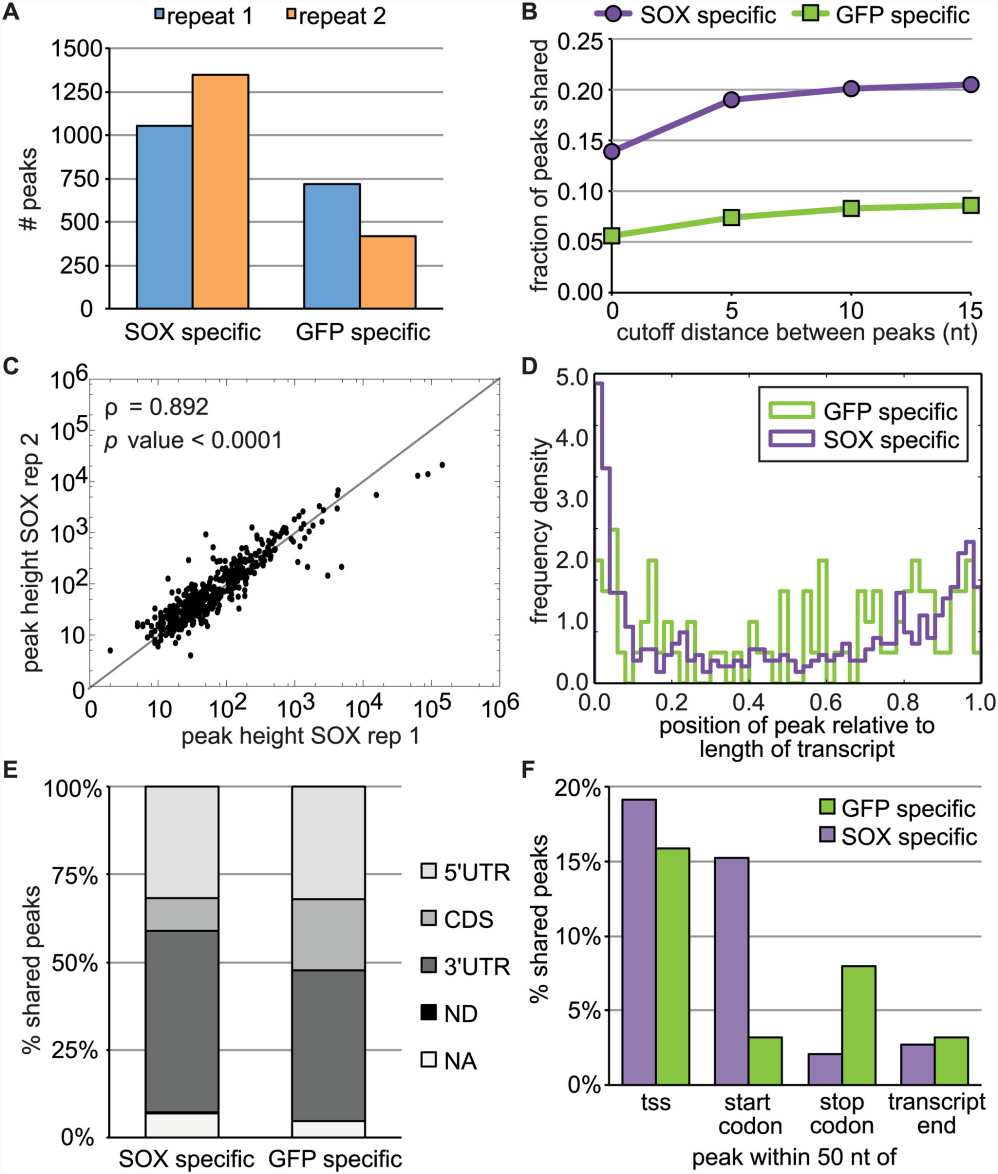
PARE analysis identifies SOX-specific cut sites in endogenous RNAs. A) Number of peaks/cut sites identified specifically in SOX or GFP samples. B) Fraction of the peaks identified in SOX or GFP samples that were detected in both biological replicates (“shared peaks”), relative to the maximum distance allowed between the peaks. C) Correlation plot of the heights of peaks found in both SOX+ samples. Peak height is defined as the highest read count within the peak window, at the position defined as the putative cut site. D) Position of the cut sites found in both replicates (“shared cut sites”) within the mRNAs relative to the length of the transcript. E) Position of the shared cut sites relative to the coding region of the mRNA (in all samples > 90% of the peaks fall within coding transcripts). ND = not determined, because multiple transcript isoforms are present in the annotation and the cut site position would differ between isoforms. NA = no coding sequence annotated. F) Position of shared cut sites relative to annotated landmarks on transcripts. For all panels in this figure, a scanning window of 4 nt, a multiplicative factor of 4, a confidence level of 99.99%, were used to predict cut sites. All cuts sites ≤ 5 nt apart in the two replicates were used for the analyses in panel C-F (SOX-specific peaks: n = 456, GFP-specific peaks: n = 84).

Using the approach detailed above, we detected a higher number of peaks specific to SOX-containing samples relative to control samples, consistent with broad mRNA targeting by SOX (Fig 2A; S2 Table). Even when varying the allowed distance between peaks from 0–15 nt in sample replicates, the SOX samples contained ~3–5 times the number of reproducible (“shared”) SOX-specific peaks (S2C Fig). Up to 20% of the SOX-specific peaks but fewer than 9% of the control GFP-specific peaks were shared between the replicates, indicating that many of the SOX cleavages reproducibly occur at a given site (Fig 2B). The read counts at the putative cut site in the two SOX replicates were highly correlated (Fig 2C, Spearman’s ρ = 0.892, *p* value < 0.0001), further demonstrating that these peaks correspond to specific SOX-mediated cleavages. For downstream analyses, we focused on cut sites that were detected in both the replicates using the 99.99% confidence level and were 0–5 nt apart (S3 Table). Example plots of the read counts around identified cut sites are shown in Figs 1C and S3A–S3F.

Several virally encoded host shutoff factors that trigger RNA degradation, including herpes simplex virus 1 vhs and SARS coronavirus nsp1, are thought to induce sequence-independent cuts near the 5’ end of the message [20,21]. To examine whether SOX cleavage sites in endogenous mRNAs are position-specific, we compared the location of the SOX-specific cut sites to those found only in control samples using the human transcript annotation from ENSEMBL GRch37. In both SOX and GFP control samples, more cut sites occur towards the ends of the transcripts, most frequently corresponding to the 5’ and 3’ untranslated regions (UTRs) of the mRNA (Fig 2D and 2E). It remains unclear whether this non-specific end bias is due to a general preference for cleavage in non-translated regions or a consequence of the PARE approach. We also computed the position of the cuts relative to landmarks such as the transcript start site, start codon, stop codon or annotated 3’ end. Only a fraction of the peaks was located within 50 nt of any of these landmarks in either case (Fig 2F). Although a greater percentage of the SOX cut sites occurred within 50 nt of start codons, this still only accounted for 15% of the cut sites. Furthermore, the 15–20% of both SOX and GFP peaks near annotated transcription start sites may represent the beginning of full-length decapped mRNAs rather than endonuclease cleavage fragments. Collectively, these analyses indicate that SOX cut sites are not restricted to a particular region of the mRNA, although cleavage sites in both SOX and control GFP samples may be enriched in areas of the transcripts that are not covered by ribosomes. These findings are consistent with our previous reporter mRNA data [5].

### Sequences around SOX cleavage sites in endogenous mRNAs contain SOX targeting elements

**Fig 3:**
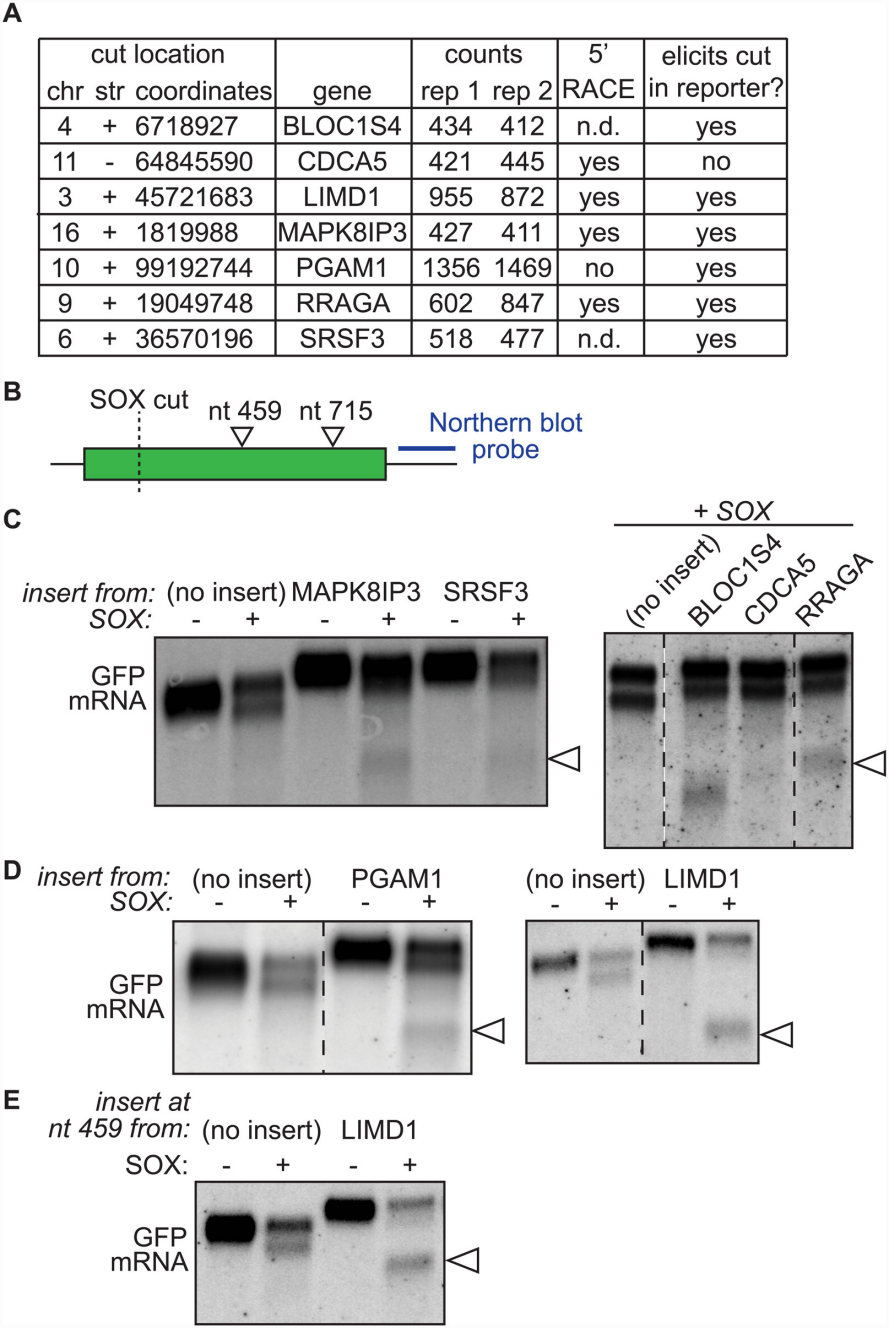
The sequences surrounding the SOX cut site in human mRNAs contain a SOX targeting sequence. A) Select SOX cut sites chosen for validation, including chromosomal position, parent gene and reproducibility by 5’ RACE and insertion experiment (“elicits cut?”). n.d. = not detected with RACE primers used. B) Diagram of the GFP construct indicating the position of inserted sequences and of Northern blot probes. C-E) 200 nt sequences surrounding SOX cleavage sites in the indicated endogenous genes were cloned into the GFP reporter at nt 715 (with the exception of panel E; in this case the insertion was at nt 459). The GFP- based reporters were then co-expressed with SOX (“+” or “+SOX”) or an empty vector control (“-”) in shXrn1-treated cells and the GFP mRNA from these cells was detected using Northern blotting. The arrowheads point to the additional cleavage fragments resulting from the insertions. Images are representative of results from at least three experimental replicates.

We next selected seven SOX cut sites for independent experimental validation (Fig 3A). The selection was based on three criteria: 1) position more than 50 nt from the annotated 5’ end of the transcript in order to eliminate potential transcription start sites, 2) high number of mapped reads, and 3) clear SOX-specific peaks in a visual inspections of the read plots (Figs 1C and S3A–S3F). We used two approaches to validate the cut sites, targeted 5’ RACE and insertion in reporter constructs, and found that all sites validated in at least one of the two assays. We detected a 5’ RACE fragment that appeared specifically in SOX-expressing cells (S3G Fig) and whose size corresponded to the predicted SOX cleavage location for four of the transcripts. (We were unable to detect the RNAs for BLOC1S4 and SRSF3 using control primers).

Our second validation approach was designed to test the hypothesis that specific RNA sequences or structures flanking endogenous cut sites direct cleavage by SOX even when removed from their native context, as we had seen for reporter mRNAs [5]. We inserted 200 nt surrounding the cleavage sites from the mRNA targets identified by PyDegradome into a GFP reporter (Fig 3B). We then co-expressed these constructs with SOX in Xrn1-depleted cells and tested whether the inserted sequence caused a SOX-specific cut in the mRNA. The GFP reporter we used is normally cut by SOX at ~nt 140 of the coding region [5], generating a degradation intermediate that is ~ 250 nt shorter than the full length mRNA. We found that the insertion of the sequences from six out of seven of the candidate SOX cleavage sites resulted in the appearance of a second RNA fragment in SOX-expressing cells (Fig 3C and 3D). Interestingly, the intensity of the additional cleavage products varied between the insertions, suggesting some sequences are better SOX targets than others. In particular, in the GFP reporters with insertions from the LIMD1 mRNA (Fig 3D), we found that the original cleavage site in the GFP coding region was almost completely abolished in favor of the new cleavage site, as evidenced by the disappearance of the longer degradation intermediate. Moreover, insertion of the LIMD1 200 nt sequence in a different position in the GFP mRNA also elicited SOX cleavage (Fig 3E), further demonstrating functionality of the targeting sequence regardless of its broader context. Taken together, these data indicate that we have identified *bona fide* SOX cleavage sites in endogenous mRNAs, and that these sites contain specific elements that lead to SOX targeting.

### The SOX cleavage site likely occurs in an unstructured region of the mRNA and is characterized by an A-rich sequence just upstream of the cleavage

**Fig 4:**
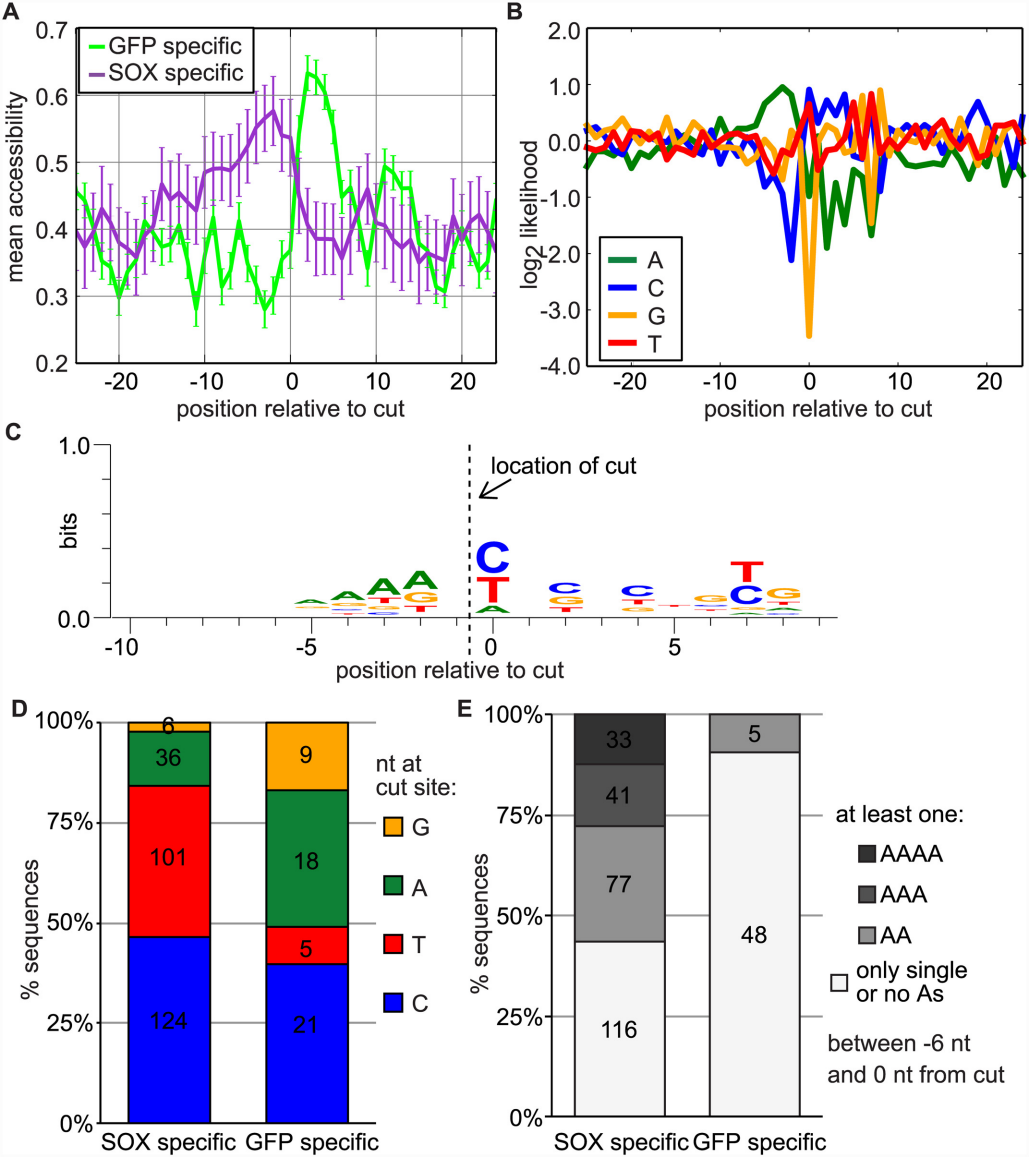
Analysis of sequences surrounding the site reveals common structural and sequence features. A) Mean accessibility (unpairedness) of the nucleotides surrounding the cut sites found in the GFP or SOX samples. The accessibility was computed using Localfold [28] on a 300 nt sequence surrounding the cut site (SOX: n = 166, GFP: n = 30). B) Log2 likelihood of each of the 4 nucleotides at position relative to the cut in cut sites shared between the two SOX samples. (Values for the background distribution of the nucleotides were computed from a list of human transcripts) (n = 261). C) Weblogo3 [42] representation of the frequency of each base in the 20 nt surrounding the cut sites found in both SOX samples (n = 267). The position of the cut site is indicated. D) Percentage of sequences with each of the four nucleotides at the cut site (position 0) among the GFP or SOX specific peaks. E) Percentage of sequences surrounding putative cut sites that contain at least one oligo-A stretch within the 6 nt upstream of the cut (for D and E, SOX: n = 267, GFP: n = 53). For all panels in this figure, the shared cut sites were determined based on a scanning window of 4 nt, a multiplicative factor of 4 and a confidence level of 99.99%. All cut sites with the same exact position in both SOX or both GFP samples that were >50 nt away from an annotated transcription start site and had sufficient surrounding sequences within the same annotated exon (300 nt in A, 50 nt in B and 20 nt in C and D) were included in these analyses. The varying number of sequences used for the analyses in the different panels is a result of the requirement for sufficient flanking sequences in the same annotated exon, but as many sequences as possible were analyzed in each case.

To identify features that define a SOX cleavage site, we searched the sequences surrounding the SOX cut sites detected in both biological repeats for structural or sequence similarities, using the cut sites shared by the two GFP samples as a comparison set. First, Localfold [28] was used to determine the likelihood that the nucleotides around the cut are located in unpaired regions (i.e. accessibility). This program is a variation on the Vienna algorithm RNAfold and is based on the assumption that potential structures are formed locally, which is consistent with the success of our insertion experiments. We found that the nucleotides 5’ of the SOX cut were more accessible (thus presumably unstructured) than surrounding sequences (Fig 4A). This pattern was different from the sequences surrounding the GFP sites, suggesting that it is feature specific to SOX cleavage sequences.

We also computed the log likelihood for different nucleotides at positions around the SOX cleavage (Fig 4B and 4C). Although no strong consensus sequence emerged, two features stood out from this analysis. First, the position right after the cut site (position 0) was preferentially C or T. When we computed the frequency of different nucleotides at the cut site for both SOX-specific and GFP-specific cleavages, we found that the pyrimidines C or T were found at 85% of the SOX cut sites, whereas C or A were the most frequent bases at position 0 in cuts specific to GFP control samples (Fig 4D). This distribution is not due to a bias in library preparation, as A was the most frequent base at the beginning of both aligned (S4A Fig) and total (S4B Fig) reads.

We also found that there were more As and fewer Cs in the 10 nt 5’ to the cleavage site. We computed the fraction of putative SOX cut sites that had A dimers or trimers in the 6 nt preceding the cut and found that A stretches were found before ~60% of our mapped cut sites (Fig 4E). This was not a general feature of the sequences that produce RNA fragments, as only five of the cut sites found solely in the control samples were preceded by an A dimer and none by longer A stretches. These analyses suggest that although SOX cut sites are defined by a degenerate sequence pattern, this sequence is enriched for pyrimidines at a cut site adjacent to an unstructured stretch of A residues.

### Experimental analysis of an endogenous SOX cleavage sequence confirms role of oligo A sequence and potential structural element

**Fig 5:**
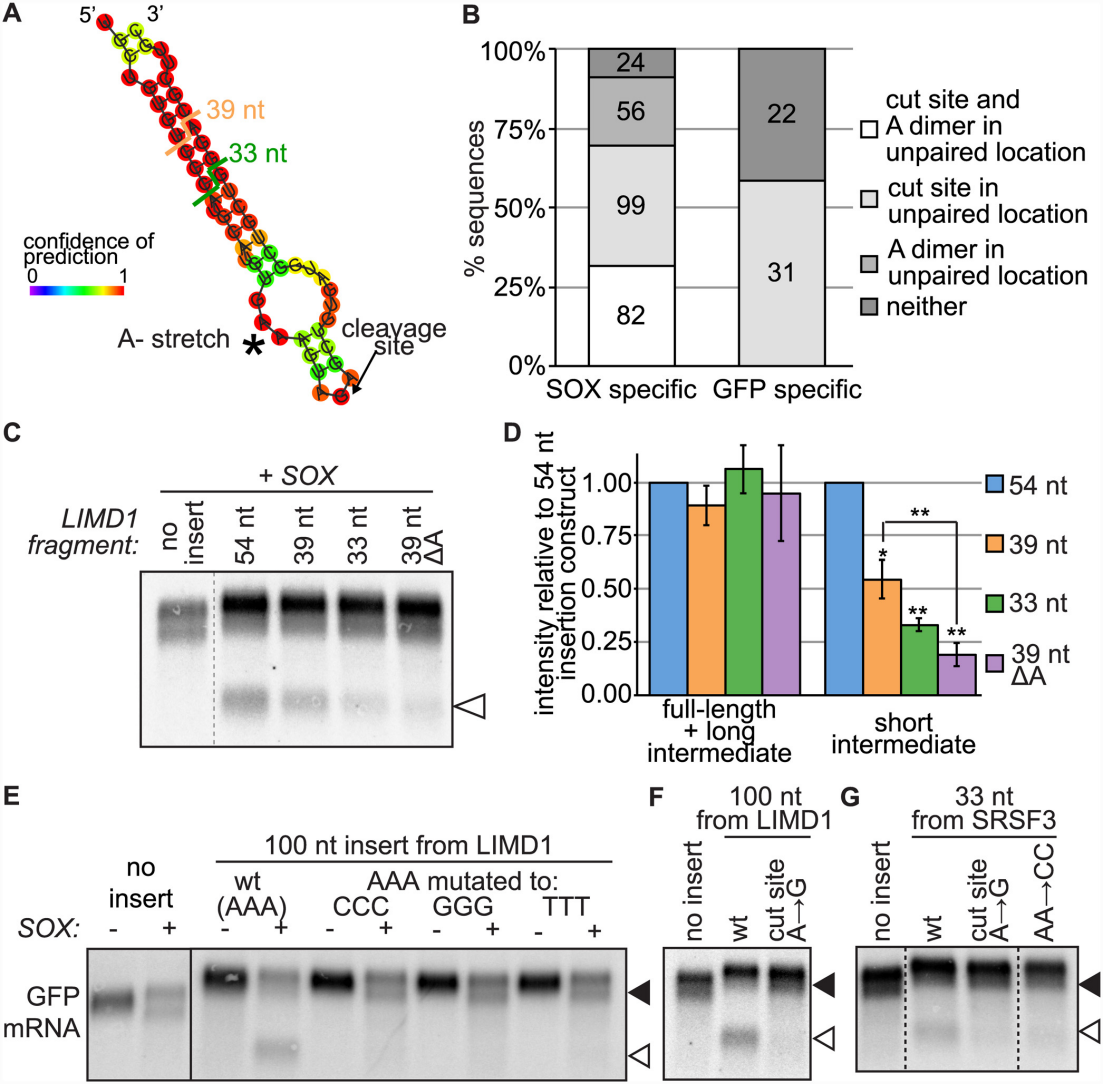
The sequence features at the SOX cut site in LIMD1 and SRSF3, as well as a structural element around the site, are required for SOX cleavage. A) Predicted structure of the 54 nt surrounding the SOX cut site in LIMD1, highlighting the A trimer (asterisk) and the cut site (arrow), as well as the ends of the 39 nt, and 33 nt insertions used in D. B) RNAfold was used to predict the structure of all 50-nt sequences surrounding SOX-specific and GFP-specific cut sites (based on a scanning window of 4 nt, a multiplicative factor of 4 and a confidence level of 99.99%). The proportion of cut site where the location of the cut was predicted to be unpaired, that had an unpaired A dimer within 5 nt of the cut or both is plotted. C-G) GFP reporters were co-expressed with SOX (“+”) or an empty vector control (“-”) in shXrn1-treated cells. The GFP mRNA was detected using Northern blotting. The empty arrowheads point to the additional cleavage fragment resulting from insertions, whereas the filled arrowheads point to the normal GFP cleavage fragment. C-D) 54–33 nt surrounding the SOX cleavage site in the LIMD1 mRNA were inserted into the GFP reporter at nt 715. In the 39 nt ΔA construct, one of the three As found at positions -7 to -5 from the cut site was deleted. A representative blot is shown (C), as well as the quantified intensity of the signal from the different RNA species (D), plotted relative to the intensity of the bands from the 54 nt insertion construct. Error bars = standard deviation, ** *p* < 0.001, *p < 0.05 (Student’s *t-*test). E) The A trimer preceding the SOX cut site in LIMD1 was mutated to a C, G or T trimer in the LIMD1 100 nt insertion construct. Images are representative of results from at least three experimental replicates. F) The A immediately 3’ of the SOX cut site in LIMD1 was mutated to a G in the 100 nt insertion construct. G) The A immediately 3’ of the SOX cut site in SRSF3 was mutated to a G (A → G) and the A dimer preceding the SOX cut site was mutated to a C dimer (AA → CC) in the 33 nt insertion construct.

To probe the SOX targeting element further we examined more in detail the SOX targeting element in the validated endogenous mRNAs (Fig 3A). The structure prediction program RNAfold [29] predicted that the 50–54 nt surrounding the cut sites in six out of seven of the RNAs fold in hairpin structures with oligo-A stretches and the cleavage sites in unpaired loops (Figs 5A and S5A). The only exception was MAPK8IP3, which also lacked the oligo-A sequence. These structure predictions mirror the accessibility results from Localfold analysis (Fig 4A), which indicated that the positions from -10 to 0 relative to SOX cut sites are more likely to be unpaired. Similarly, we predicted the structures of all 50 nt sequences surrounding the SOX-specific cut sites in our dataset and determined how many of the sequences presented either the cut site, an A dimer or both the cut site and an A dimer in an unstructured region. For 90% of the SOX cut sites, at least one of these two features was predicted to be in an unpaired region, with over 30% of the sequences predicted to have both (Fig 5B).

We reasoned that if these structural features are important for cleavage, the efficiency of the cleavage could vary depending on the length of the inserted endogenous sequence. Fragments of different sizes may not be able to fold into the native structure equally well and the stability of the resulting structures may vary. Consistent with this idea, we found that changing the length of the inserted fragments for two different RNAs (LIMD1 and SRSF3) changed the efficiency of SOX cleavage, measured by the intensity of the degradation intermediate (Figs 5C, 5D, S5B and S5C), although sequences of 33 nt were sufficient to elicit SOX cleavage. In particular, when we shortened the LIMD1 inserted sequence from 200 nt to 54 nt, 39 nt and 33 nt, the efficiency of cleavage progressively diminished (Fig 5C and 5D), consistent with LIMD1 sequences adopting a stem-loop structure that becomes destabilized upon sequential deletions of the putative stem region. Surprisingly, shortening the inserted sequence for SRSF3 had the opposite effect and increased the efficiency of the cleavage (S5B and S5C Fig). Because the same sequences are present in the 200 nt and the 33 nt SRSF3 insertion, this observation cannot be explained by the presence of a targeting sequence alone. Instead, we hypothesize that the shorter insertion folds more stably into an autonomous element that is required for targeting by SOX.

We previously found that mutating a TGAAGT sequence 5 nt before the GFP cut site to TGAGTG could abolish the cleavage site in GFP (S6A Fig). The LIMD1 site is also preceded by a similar TGAAAG sequence predicted to be in an unpaired loop. To test directly whether the A trimer in the LIMD1 sequence was required for the positioning of the cleavage, we mutated the TGAAAG sequence in our insertion reporter to TGCCCG, TGGGGG or TGTTTG. As predicted by our data analysis, we found that mutation of the A trimer prevented the LIMD1 sequence from eliciting SOX cleavage, indicating that the AAA is an integral part of the SOX recognition site (Fig 5E). Moreover, deletion of one of the three As in the 39 nt insertion reduced the efficiency of cleavage dramatically (Fig 5C and 5D), while RNAfold structure prediction suggested that this deletion is unlikely to substantially alter the structure of the RNA (S6B Fig). Similarly, mutating an A dimer just upstream of the cut site in a SRSF3 insertion construct reduces SOX-mediated cleavage (Fig 5G). While the upstream A dimer likely contributes to SOX targeting, we note that it is not always required, as a similar mutation in a PGAM1 insertion construct did not abolish cleavage (S6C Fig). Overall, these data are consistent with the idea that the A-stretch is an important feature of the SOX cleavage specificity.

Lastly, our analysis indicated that the nucleotide G is under-represented at the position immediately following the SOX cut site (position 0, Fig 4B–4D). We found that mutating the nucleotide at position 0 from an A to a G prevented SOX-mediated cleavage in two out of three of the RNA we tested (LIMD1 and SRSF3, but not the PGAM1) (Figs 5F, 5G and S6D). These data suggest that SOX activity is inhibited by the presence of a G nucleotide as the residue 3’ of the cleavage. Collectively these data experimentally validate our computational finding and strongly suggest that SOX cut sites are defined by a combination of sequence and structural features.

### A conserved sequence pattern is specific to SOX-dependent cut sites

**Fig 6:**
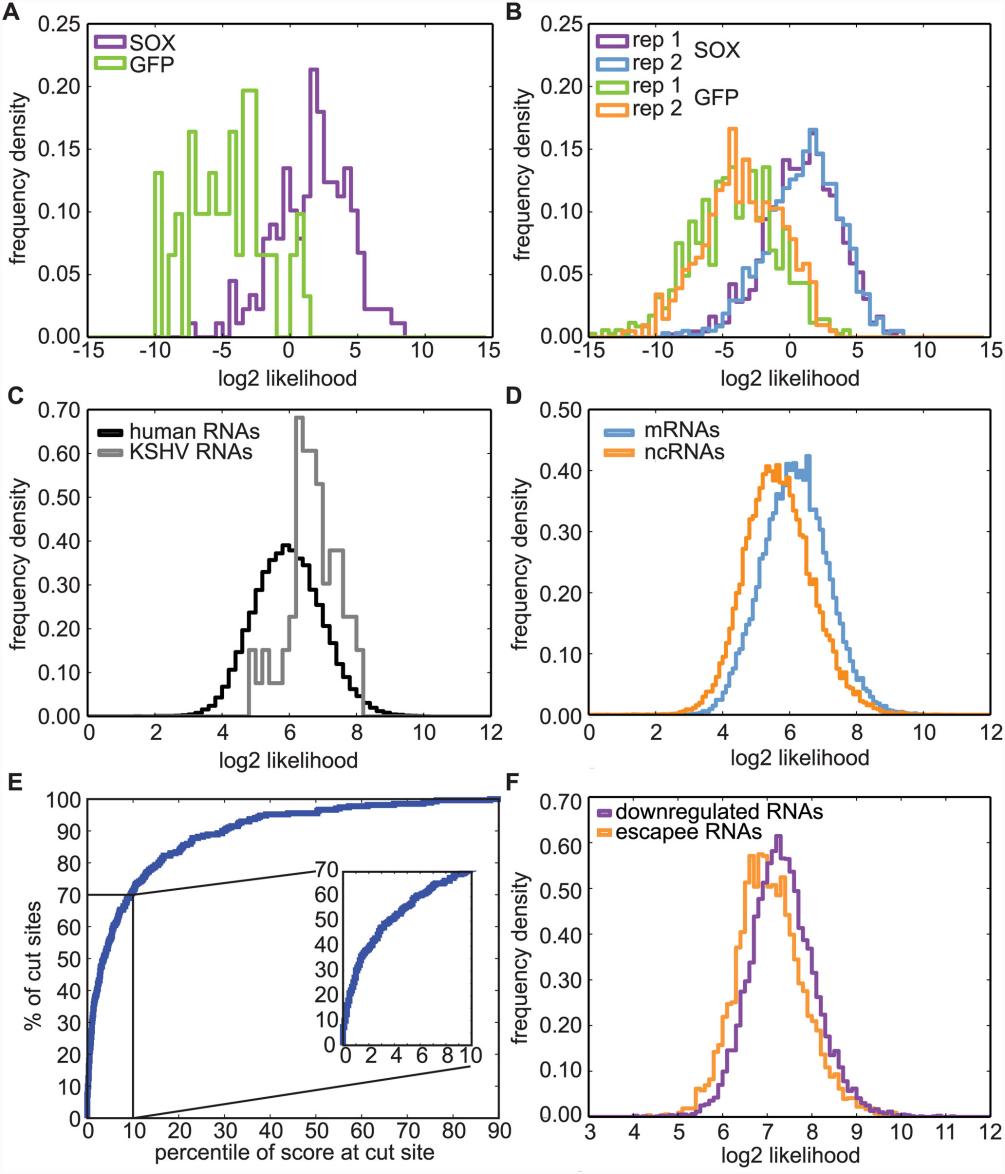
A degenerate motif defines SOX cut sites. A position weight matrix (PWM) for nucleotide likelihood in the 20 nt surrounding the SOX cut sites was generated from the 129 sequences that contained SOX-specific sites with a confidence level of 99.999%. Sequences were scored using this matrix, after removing the 129 “parent” sequences where applicable. A) Frequency distribution histogram of log likelihood scores for the 20 nt surrounding the GFP- or SOX-specific cut sites. GFP: n = 61; SOX: n = 178. B) Frequency distribution histogram of log likelihood scores for the 20 nt surrounding all cut sites found in the two GFP and SOX samples. GFP rep1: n = 693; GFP rep2: n = 409; SOX rep1: n = 849; SOX rep2: n = 1160. C) All human and KSHV annotated transcripts were scored using the PWM. The frequency distribution histogram for the top scores for each transcript is plotted. D) Human transcripts were divided into coding and non-coding based on the annotation in ENSEMBL. The frequency distribution histogram for the top scores for each transcript in the two sets of human RNAs is shown. *p* value (Kolgorov-Smirnoff test) < 0.001. E) All possible scores for mRNAs carrying an observed SOX cut site far from the transcription start site (n = 271, confidence level = 99.99%) were computed and ranked in comparison to all the possible scores for the transcript containing the cut. 10 out of 271 (4%) of the cuts were found at the site with the best score. The cumulative frequency distribution for the percentile of the score at the cut was plotted. F) Human RNAs were classified into SOX targets (“down-regulated RNAs”) or SOX escapees (“escapees RNA”) based on the results from Clyde and Glaunsinger [7]. The frequency distribution histogram for the top scores for each transcript in the two sets is shown. *p* value (Kolgorov-Smirnoff test) < 0.001.

Although sequences flanking the SOX cleavage sites lacked a strong consensus motif, our analysis showed that the frequency of the bases around the cut site diverged from the expected distribution for human RNA sequences (Fig 4B and 4C). This suggested to us that there is a conserved sequence pattern among SOX target sequences. In order to be able to search RNA sequences for this variable motif, we derived a position weight matrix (PWM) for the positions -10 to +9 from the SOX cleavage site (0 being the nucleotide 3’ of the cut site, Fig 4C). The PWM is a matrix that lists the probabilities (transformed into log likelihood to correct for the background frequencies of the nucleotides) for each of the four bases at each of the positions of the putative motif. The log likelihoods for our PWM were derived from 129 very high confidence SOX cut sites identified in both of our experimental repeats using a very stringent confidence level of 99.999% and located at least 50 nt away from a transcriptional start site. The logo in S7A Fig is a pictorial representation of the PWM. We then used the PWM to confirm that SOX cut sites specifically matched the motif by scoring several subsets of potential SOX target or control sequences using the PWM. The presence of preferred nucleotides in positions -10 to +9 from the cut site results in higher (positive) log likelihood scores, whereas a poorer match to the motif produces a lower (negative) log likelihood score. Indeed, the sequences flanking the set of reproducible SOX cut sites (identified with a confidence level of 99.99%) were a closer match to the motif compared to those surrounding GFP-specific fragment ends, as shown by the distribution of the log likelihood scores (Fig 6A). A similar difference was seen when analyzing the sequences surrounding all potential SOX and control (GFP) cut sites from each of the two biological repeats. Sequences around SOX cut sites were a good match (positive log likelihood score) to the motif more frequently than control sequences (Fig 6B). In both analyses (Fig 6A and 6B), we removed the sequences of the 129 high confidence cut sites used to derive the PWM from the analyzed sets. The results of these analyses indicate that although the precise sequence composition may vary, there is a specific element marking SOX cut sites that is not observed in control samples. Furthermore, our stringent analysis parameters have likely resulted in an underestimation of the number of true SOX targeting sites, because even sites detected in only one of our two replicates were generally a good match to the SOX targeting motif.

### The cleavage site motif is common among human and viral transcripts and correlates with SOX-mediated transcript degradation

We next used the PWM to test whether the prevalence of the targeting element differed between the human and viral mRNA transcriptomes. We analyzed annotated transcripts with a 20 nt scanning window, computed a log likelihood score for every possible 20 nt sequence, determined the highest possible motif score for each transcript and plotted the distribution of the scores. In agreement with the widespread mRNA cleavage by SOX, most of the annotated human transcripts have at least one sequence that is a good match to the motif (log likelihood score > 0, Fig 6C). The prevalence of high scoring sequences may explain how SOX is able to degrade most transcripts. Moreover, we found that KSHV transcripts also contained sequences that matched the motif (Fig 6C), which suggests that sequence specificity is not used by SOX to discriminate between host and viral mRNAs. This results is consistent with findings from the related gamma-herpesvirus MHV68 [16] that show degradation of viral transcripts by proteins of the SOX family. We have listed examples of human and KSHV RNAs with high log likelihood scores in S4 Table. Interestingly, within human transcripts, coding RNAs tended to contain sequences that were slightly better matches to the motif than non-coding RNAs (ncRNAs, Fig 6D), as did spliced RNA in comparison to non-spliced RNAs (S7B Fig). The meaning of these small but statistically significant differences is unclear, particularly since the log likelihood scores for all groups were generally high, indicating the presence of good sequence matches to the motif.

We also wanted to test whether the sequences around the experimentally determined SOX cleavage sites were more likely to match the motif than other locations on the same transcripts. We analyzed the transcripts containing SOX cuts sites identified in the PARE data using a 20-nt scanning window as described above. We then ranked the log likelihood scores for all possible 20-mers in the transcript, and asked how highly ranked the score for the actual cut site was. 70% of the experimentally observed cut sites ranked within the top 10% of scores for their RNA, indicating that the surrounding sequences were a very good match to the motif compared to other sites in the same mRNA (Fig 6E).

Finally, we tested whether the relative degradation of different human transcripts by SOX was correlated with how well their sequences matched our degenerate motif. Human transcripts were classified as down-regulated by SOX or SOX escapees based on the data from an RNAseq experiment comparing human mRNA levels in cells overexpressing GFP-SOX or GFP alone [7] The best motif score for each RNA detected in the RNAseq experiment was then computed using a 20-nt scanning window as described above. We found that the down-regulated RNAs (fold change < 0.75) had better motif scores than the escapee RNAs (Fig 6F). Similarly, when we plotted the fold change for each gene against the best motif score for that gene, we found that there was a modest but significant inverse correlation between the fold change in mRNA levels and the motif scores (Spearman’s ρ = -0.21. *p* value < 0.0001, S7C Fig).

These analyses suggest that the level of down-regulation of mRNAs by SOX is in part determined by the degree to which their sequence is a good match for the SOX targeting motif.

## Discussion

We have identified key sequence features of the targeting element that directs the RNA endonuclease SOX to cleave a significant fraction of the mRNA transcriptome. As we had hypothesized from the analysis of individual reporter mRNAs [5], SOX cleavages in endogenous mRNAs occur in a sequence-specific manner. Although surprisingly large, this element is not defined by strong sequence consensus, but instead contains a small number of conserved residues. Structural features may also contribute to motif-driven SOX targeting.

Our data resolve how a sequence-specific nuclease can target such a breadth of targets. SOX presents a model of RNA targeting in which cleavages are at the same time sequence specific and highly promiscuous. This is achieved through the use of a degenerate sequence/structure pattern that is anchored by key residues to define specific RNase targeting locations. Good matches for a loosely defined sequence pattern can be found in all viral and host RNAs, enabling cleavage to be simultaneously specific and widespread. Although this approach may be less efficient than the location-driven targeting of the cap-proximal region reported for other host shutoff factors that promote mRNA cleavage [20,21], such a mechanism may provide more regulatory opportunities. Also, it may explain why SOX has less dramatic effects on RNA than other viral RNases [3]. Many cellular endonucleases have few described targets and transcriptome-wide targeting analyses of other cellular and viral RNases are limited. It will thus be of interest to apply high-throughput sequencing approaches to isolate degradation fragments in other systems and investigate whether any other viral or cellular RNases use principles similar to those employed by SOX to achieve target specificity. Notably, recent studies of specificity of RNase L, a host RNase that is activated in response to viral infection and cleaves viral RNAs and host rRNAs, have also suggested that a combination of sequence and structure is important for targeting [30,31]. However, the requirements for RNase L targeting appear less stringent than those for SOX, as the preferred cleavages sites occur at UU/UA dinucleotides in unpaired regions of structured RNAs [30,31].

The RNA motif underlying SOX cleavage specificity could be used for target selectivity, enabling a subset of viral and cellular mRNAs to escape cleavage. Our observation that the majority of both viral and cellular transcripts contain the SOX targeting motif is in agreement with the fact that in MHV68, viral mRNAs are broadly susceptible to degradation by the SOX homolog muSOX [16]. However, subsets of viral and cellular transcripts are not susceptible to host shutoff [6,7,16,17], and it is likely that at least some of these escape due to the absence of a robust targeting element. Indeed, the correlation between the scores of matches to the motif and the level of degradation of host mRNAs suggest that sequences within the motif can influence rates of degradation of different RNAs. This may result in a more nuanced effect of SOX-mediated degradation. We also note that the level of degradation seen by steady-state level measurements is likely influenced by additional variables that are unrelated to the efficiency of SOX cleavage, including the efficiency of removal of different sequences by Xrn1 and a reduction of transcriptional rate due to a feedback loop triggered by the RNA degradation [32]. Therefore the relationship we see may be an underestimation of the contribution of targeting preference. An additional level of regulation for transcript selectivity is also provided by the presence of dominant protective elements like the SOX-resistance element we have identified in the 3’ UTR of the IL-6 gene [8,9], which prevents cleavage of the GFP RNA despite the presence of a strong targeting sequence. We expect that both dominant and passive mechanisms of escape from SOX-mediated targeting ultimately shape the landscape of host gene expression during SOX-mediated host shutoff.

A limitation of our analyses is that we are unable to readily explore, both computationally and experimentally, the contribution of structural elements to the SOX cleavage site. Computational analysis of shared structures is difficult when the sequences involved are not evolutionarily related. Moreover, even in well-characterized examples of the same protein binding different RNAs, for example in the case of bacterial ribosomal proteins binding both ribosomal RNAs and their own 5’ UTRs, the features that are recognized are highly variable [33], and the remainder of the structure may serve as a scaffold. Nonetheless, our identification of endogenous cut sites makes experimental analysis of a putative SOX target structure possible in the future.

A major outstanding question is how SOX recognizes the targeting motif. *In vitro* studies indicate that the binding affinity of SOX for RNA is much lower than its affinity for DNA [34], which is also processed by SOX during viral genome replication in the nucleus. This suggests that SOX may not recognize RNA targets directly but may instead be recruited by a protein partner. This model is supported by the observation that point mutations that abolish SOX host shutoff activity in cells do not affect its RNase activity *in vitro* [34,35], pointing at a likely protein-protein interaction. A SOX partner protein would have to be a fundamental factor in RNA metabolism and/or a RNA binding protein with promiscuous specificity, as it must bind a large portion of the cellular RNAs. Another possibility is that SOX directly recognizes its target sequence, and that the apparent low affinity for RNA *in vitro* is due to the fact that a non-cognate sequence was used for the binding assay. However, the fact that SOX cleaves the GFP RNA sequence when GFP is expressed from an RNA polymerase II promoter, but not when it is expressed from an RNA polymerase I or III promoter [5] argues against this scenario. How this motif potentiates SOX targeting, as well as whether it is used as a protein-binding scaffold for other purposes in the cell related to mRNA fate remain important questions for the future.

## Materials and Methods

### Cells and Xrn1 knockdown

HEK293T, HEK293T shXrn1 and KSHV-infected iSLK-219 [36] and iSLK-219 shXrn1 cells were maintained in high-glucose DMEM (Gibco) supplemented with 10% fetal bovine serum (Hyclone). shXrn1 cells were generated using pTRIPZ-shXrn1 (Thermoscientific, clone V2THS_89028/RHS4696-99704634, targeting sequence: TATGGTGAGATATACTATG). To induce expression of the shXrn1, cells were treated with 1 μg/ml doxycycline (Fisher) for 4–5 days prior to harvesting. Lytic induction of KSHV was induced in iSLK-219 cells [36] by treating cells with 1 μg/ml doxycycline and 1 mM sodium butyrate for 4–5 days prior to harvesting. The same induction also led to anti-Xrn1 shRNA expression in the iSLK-219 shXrn1 cells. For the second biological repeat of the PARE experiment, cells were transfected twice with the siRNA against Xrn1 as previously described [5].

### Plasmids

pd2eGFP-N1 was purchased from Clontech. pCDNA3.1-GFP-SOX was previously described [7]. pCDEF3-SOX was previously described [13]. pd2eGFP-ΔTGAAG was previously described [5]. To test SOX-mediated cleavage of endogenous mRNA sequences, 33–200 nt surrounding putative cleavage sites in the human mRNAs to be tested were cloned from HEK293T cDNA using Vent polymerase (NEB). They were then inserted into the BsrGI site at nt 715 of the GFP coding region in pd2eGFP-N1, as previously done to test GFP sequences [5], either by restriction enzyme digest or through a modified version of Quikchange mutagenesis [37]. An EcoRV site was also generated at nt 459 of the GFP coding region using Quikchange mutagenesis (Agilent) and the 200 nt surrounding the cleavage site in LIMD1 were inserted at this location. Quikchange mutagenesis (Agilent) was used to insert out the following mutations: 1) mutate the AAA sequence preceding the LIMD1 cleavage site to CCC, GGG and TTT in the pd2eGFP construct containing the 100 nt LIMD1 fragment; 2) mutate the AA sequence preceding the cleavage site to CC in the pd2eGFP constructs containing either the 100 nt PGAM1 or the 33 nt SRSF3 fragments; 3) mutate the cleavage site from A to G in the pd2eGFP constructs containing either the 100 nt LIMD1 fragment, the 100 nt PGAM1 fragment or the 33 nt SRSF3 fragment; 4) mutate the GFP TGAAGT to TGAGTG. All primers used for cloning are listed in S5 Table.

### PARE library preparation and sequencing

HEK293T cells treated with siRNAs against Xrn1 (repeat 1) or expressing shRNAs against Xrn1 (repeat 2) were transfected either with pCDNA3.1-GFP-SOX or pd2eGFP-N1. In both cases >75% transfection efficiency was observed. One day after transfection total RNA was harvested and purified using RNABee (Teltest). RNA was then treated as described in Zhai et al. [24] to generate PARE libraries. Briefly, poly(A)+ RNA was purified, and RNA adapters were ligated to free 5’ phosphate-bearing RNA ends. A second poly(A) purification was used to remove unligated adapter. cDNA was synthesized using oligodT directed primers, and the cDNA was then amplified 15 times. As the adapter includes an MmeI restriction endonuclease site, MmeI was used to cut the double stranded amplicons 20 bp downstream of the adapters. 3’ dsDNA adapters were then ligated to the 3’ end of the amplicons. This created libraries of 20 bp tags corresponding to the 5’ end of RNA fragments flanked by adapters, similar to small RNA libraries. Libraries were checked on an Agilent Bioanalyzer and sequenced at the Vincent J. Coates Genomics Sequencing Laboratory at UC Berkeley using a HiSeq2000 Illumina sequencer. Raw data are available on the NCBI Gene Expression Omnibus database as study GSE70373.

### Reads preprocessing and alignment

Reads flagged by the CASAVA 1.8 program were eliminated and cutadapt [38] was used to trim away the adapter sequence at the read 3’ end (sequence: TGGAATTCTCGGGTGCCAAGGAACTCCAGT). Because the PARE protocol should produce 20–21 nt sequence tags from the 5’ ends of phosphorylated RNA fragments, trimmed reads that were longer than 22 nt or shorter than 19 nt were discarded. The resulting sequences were aligned using Tophat 2.0.10 [39] using bowtie1 as recommended for short sequences. No mismatches were allowed (-N 0 option), and only alignments that uniquely mapped to the annotated portion of the genome (-T -x 1 options) were retained, to simplify downstream analysis. For the alignment and subsequent analyses, GRch37 and the ENSEMBL annotation for this genome build were used. These and other analysis were carried out on an iMac computer (Mid 2011 model, 3.4 GHz Intel Core i7, 16GB RAM).

### PyDegradome (Python-based peak finding using Bayesian probability)

A Bayesian probability framework was used to find peaks that were specific to test samples compared to control samples, which takes into account random variations in the observed number of reads. At a given location and a given experiment, we assume that there is an underlying rate at which reads are produced, and the observed count follows a Poisson distribution with mean equal to this rate.

In both the control and test data sets, we find that the frequency of reads per location follows a power-law distribution, as is typical for gene expression and deep sequencing data [40,41], and we therefore assume that the prior distributions for the underlying rates follow this power-law distribution, where the power is fitted from the data. At a given location, we then use Bayes' theorem to construct posterior distributions for the rates, given the observations of the read counts. We then deem that there is a significant difference between the control and test at that location, if the posterior probability of the test rate being a multiplicative factor larger than the control rate exceeds some confidence level. The multiplicative factor (ratio) and confidence level are chosen by the user. The observed counts vary over a large range, from single digits up to values in the millions, and a key feature of the method is that is can effectively deal with these variations within a unified theoretical framework.

In practice, for a given control read count, we can compute a threshold for the test read count, beyond which the difference in underlying rates is significant. The software builds a table of the thresholds using bicubic splines so that many locations can be tested efficiently. The peak finding python scripts are attached as S1 Files.

Parameters were empirically optimized for the analysis so that a scanning window of 4 nt, a multiplicative factor between test/control read counts of 4 and a confidence level of 99.99% or 99.999% were used to output specific peaks. Parameters used in the different analyses are specified in the figure legends. After identifying peaks in single test/control comparisons, the peaks found in the biological repeats were compared. For subsequent bioinformatics analysis of sequences only peaks that were found in both biological repeats were used (Figs 1D, 2B, S2C).

### Motif generation and scoring

Sequences surrounding cleavage sites as defined by chromosomal positions were extracted, using the human genome assembly GRch37 build as a reference. As many sequences as possible were used for each analysis. However, because the short reads do not provide information about mRNA isoform and splicing, for all sequence analyses only cut sites that had sufficient flanking sequences within the same annotated exon were used. For motif analysis (Figs 4B, 4C and S7A) and RNAfold structure prediction (Fig 5B) 25 nt or 10 nt on either side of the cut site were used. For the accessibility computation (Fig 4A), Localfold.pl [28], a modification of the RNAplfold algorithm within the Vienna RNA package (v2.1.6) [29] was used using default settings (window = 200 nt and maxspan = 150 nt) and sequences of 300 nt on each side of the cut site were analyzed. The log likelihood of each base at each position was calculated using background frequencies of nucleotides derived from the human cDNA list from the ENSEMBL GRch37 build. Weblogo3 [42] was used to generate a graphical representation of the sequence motif from aligned sequences. To score matches to the motif, a position weight matrix was generated using log likelihoods for positions -10 to +9 relative to the cut site using 129 cut sites that were deemed high confidence in our analysis (i.e. identified using a 99.999% confidence level and position at least 50 nt away from an annotated transcription start site). The log likelihood score was then calculated for all sequences surrounding cut sites that were identified in different subsets of the data. The 129 cut sites used to generate the matrix were always eliminated from the sets that were analyzed. To compute the score matches in human and KSHV mRNA, the log likelihood score for each 20 nt sequence was calculated in all sequences longer than 20 nt listed in the human cDNA fasta repository associated with the ENSEMBL Grch37 build or in a KSHV mRNA fasta-formatted list (compiled using data from Arias et al. [43]). The highest score was recorded for each mRNA. To separate the human RNAs into coding and non-coding their ENSEMBL annotation was used. RNAs annotated as “protein coding", "nonsense mediated decay" and "non stop decay" were considered coding, whereas RNAs annotated as "antisense", "lincRNA", "miRNA", "snoRNA", "processed transcript", "unprocessed pseudogene", "pseudogene", "transcribed unprocessed pseudogene", "transcribed processed pseudogene", "processed pseudogene", and "unitary_ pseudogene" were considered non-coding. The ENSEMBL annotation was also used to determine whether the transcripts were spliced. The motif scores for human mRNAs detected in Clyde and Glaunsinger [7] were also compared to the level of degradation, that is the fold change in steady-state mRNA levels between GFP-expressing and GFP-SOX-expressing samples in the cited study. For the analysis in Fig 6F, the transcripts were categorized into “down-regulated” (fold change in SOX vs. GFP < 0.75) and “escapees” (fold change in SOX vs. GFP > 0.75). The structure of the sequences surrounding the validated cut sites was predicted using the RNAfold webserver (Vienna RNA package [29]). RNAfold v. 2.1.6 [29] was used to predict structures around all candidate cut sites, and the results were analyzed to determine whether either of the nucleotides at position -1 and 0 was predicted to be unpaired. They were also analyzed to determine whether they had an A dimer within 5 nucleotides 5’ of the cut site that was also predicted to be unpaired.

Custom Python2.7 scripts (S2 Files) were used unless otherwise noted. Where noted in the figure legends, the Kolgorov-Smirnoff test was used to determine whether the distribution of scores were significantly different.

### RNA analysis

Total cellular RNA was isolated for Northern blotting using Trizol (Life Technologies). RNA was separated on formaldehyde gels (1x MOPS buffer, 1.8% agarose, 12.3 M formaldehyde) in MOPS buffer (40 mM MOPS (Sigma), 10 mM sodium acetate, 1 mM EDTA, pH 7.0) and transferred by capillary blotting onto nitrocellulose membrane (Bio-rad) using 10x SSC buffer (1.5 M NaCl, 0.15 M sodium citrate, pH 7.0). Northern blots were probed with ^32^P-labeled DNA probes made using Decaprime II (Ambion), against the 3’ UTR of the GFP reporters. Blots were imaged using a Fujifilm scanner FLA-9000. Quantification of the blots was carried out using ImageJ [44]. 5’ rapid amplification of cDNA ends (RACE) was carried out on 5 μg of total RNA using the First Choice RLM-RACE kit following manufacturer’s protocol (Life Technologies). 5’ RACE primers are listed in S5 Table.

### Protein harvesting and western blotting

Protein was isolated for western blots in protein lysis buffer (10 mM Tris pH 8.0, 150 mM NaCl, 1% Triton X-100) containing cOmplete EDTA-free protease inhibitors (Roche), separated on SDS-PAGE gels run in Tris-glycine buffer and transferred onto PVDF membranes (EMD Millipore). Western blots were performed with mouse anti-Xrn1 antibodies (Bethyl laboratories or Santa Cruz Biotechnology, 1:1000) or mouse anti-tubulin antibodies (1:3000, Sigma Aldrich). Secondary antibodies were used at 1:5000 dilution and purchased from Southern Biotech.

## Supporting Information

S1 Fig The GFP reporter is cut in KSHV-infected cells at the same position as in SOX-expressing cells. 5’ RACE analysis of GFP degradation fragment was carried out in KSHV-infected iSLK-219 cells also expressing shRNAs against Xrn1. Reactivation of the KSHV lytic cycle was induced by addition of sodium butyrate (NaBu) and doxycycline (dox). Doxycycline addition also induced expression of an shRNA against Xrn1. A) PCR amplification product of the 5’ RACE protocol detected in the lytically reactivated cells B) Mapping of the 5’ end of the GFP RNA fragment mapped to the GFP coding region. Arrows indicate the 5’ ends detected by sequencing of the cloned 5’ RACE product, with the number of individual clones that mapped to each nucleotide. Empty arrowheads indicate positions of GFP fragment 5’ ends in transfected 293T cells from Covarrubias, Gaglia et al. [5]. Nucleotide numbering starts from the GFP ATG.
(EPS)

S2 Fig Optimization of parameters for PyDegradome. A) Western blots showing levels of Xrn1 proteins in the samples used for PARE. In repeat 1, inducible shRNAs were used, whereas in repeat 2 siRNAs were used for the knockdown. Tubulin is included as a loading control. B) The number of cut sites detected in the indicated comparisons is plotted against the parameters varied. C) Number of peaks that were identified in both replicates of SOX or GFP samples, as a function of the maximum distance allowed between the peaks. (Analysis parameters: confidence level = 99.99% and multiplicative factor = 4).
(EPS)

S3 Fig 5’ RACE detects SOX-dependent RNA fragments consistent with the deep-sequencing results. A-F) Plots of read counts (5’ ends only) for 200 nt surrounding the chromosomal position of selected SOX cut sites in the four samples. All plots represent a section of the marked exon. Note that y-axis has a logarithmic scale. G) 5’ RACE was carried out on RNA isolated from cells overexpressing SOX (“+”) or an empty vector (“-”) and treated with shRNA against Xrn1. Primers for four human RNAs detected fragments consistent with the cut sites predicted by the PARE analysis. Primers for PGAM1 did not replicate the deep sequencing data. Red asterix = expected position of RACE fragment from cleaved mRNA.
(EPS)

S4 Fig Representation of different nucleotides at the 5’ end of PARE reads. The percentage of aligned reads (A) or of the filtered reads (B) that started with each of the four nucleotides was computed.
(EPS)

S5 Fig Analysis of structures surrounding SOX cut sites in validated RNAs. A) Predicted structure of the 50 nt surrounding the SOX cut site in the validated targets, highlighting the A trimer (asterisk) and the cut site (arrow). For SRSF3, the end of the 33 nt insertion used in B-C is marked. B-C) 200–33 nt surrounding the SOX cleavage site in the LIMD1 mRNA were inserted into the GFP reporter at nt 715. The GFP reporters were co-expressed with SOX (“+”) or an empty vector control (“-”) in shXrn1-treated cells. The GFP mRNA was detected using Northern blotting. The empty arrowheads point to the additional cleavage fragment resulting from insertions, whereas the filled arrowheads point to the normal GFP cleavage fragment. A representative blot is shown (B), as well as the quantified intensity of the signal from the different RNA species (C), plotted relative to the intensity of the bands from the 200 nt insertion construct. Error bars = standard deviation.
(EPS)

S6 Fig Role of oligo-A stretch and A at position 0 in directing SOX cleavage in GFP, LIMD1 and PGAM1 RNA sequences. A) The TGAAGT sequence at positions -6 to -1 relative to the cut site in the GFP RNA (see S1B Fig) was either partially deleted (ΔTGAAG) or mutated to TGAGTG. The wild-type GFP and the two mutated reporters were co-expressed with SOX (“+”) or an empty vector control (“-”) and the GFP mRNA was detected using Northern blotting. The arrowhead points at the position of the normal GFP cleavage fragment. B) Predicted structures (with RNAfold) of the 39 nt surrounding the SOX cut site in LIMD1, showing the wild-type sequence on the left (“39 nt”) and the mutated sequence lacking one of the As on the right (“39 nt ΔA”). C-D) GFP reporters were co-expressed with SOX (“+”) or an empty vector control (“-”) in shXrn1-treated cells. The GFP mRNA was detected using Northern blotting. The empty arrowheads point to the additional cleavage fragment resulting from insertions, whereas the filled arrowhead in E points to the normal GFP cleavage fragment. The A dimer preceding the SOX cut site in PGAM1 was mutated to a C dimer (C) or the A at position 0 was mutated to G (D) in the PGAM1 100 nt insertion construct.
(EPS)

S7 Fig Additional analyses of prevalence of the degenerate SOX targeting motif. A) Weblogo3 [42] representation of the frequency of each base in the 20 nt surrounding the cut sites found in both SOX samples using confidence level setting of 99.999% and excluding sites near an annotated transcription start site (n = 129). B) Human transcripts were divided into “spliced” and “not spliced” based on the annotation in ENSEMBL. The frequency distribution histogram for the top scores for each transcript in the two sets of human RNAs is shown. *p* value (Kolgorov-Smirnoff test) < 0.001. C) The fold change in mRNA levels in SOX-expressing vs. control cells from Clyde and Glaunsinger [7] is plotted against the best motif score for that gene. Spearman’s ρ = -0.21, *p* < 0.001.
(EPS)

S1 Table Number of reads obtained from PARE. % mapping (no restrictions) indicates the percentage of reads that map to the human genome if the requirement for unique mapping to a previously annotated region of the genome is removed.
(DOCX)

S2 Table Number of peaks detected. The number of peaks detected by using each of the samples as test or control in the PyDegradome program (and plotted in Fig 2A) is listed. Parameters used for this analysis were a scanning window of 4 nt, a multiplicative factor of 4, a confidence level of 99.99%.
(DOCX)

S3 Table SOX cut sites identified by our analysis. This table lists SOX cut sites identified in both replicates with confidence level of 99.99% or in one with confidence level 99.99% and in the second with confidence level 99.9%, and that were ≤ 5 nt apart in the two replicates. The table includes the chromosomal position of the cut site, the read count at the cut site in each replicate, the gene name and the confidence level setting used for the identification. It also indicates whether the cut site could be a transcriptional start site (TSS) and whether the cut site was used for the analyses in Figs 2 and 4 or to generate the PWM for analyses in Fig 6. Only cut sites identified in both replicates with confidence level 99.99% were used for the analyses shown in Figs 2 and 4.
(XLSX)

S4 Table List of human and KSHV transcripts with highest log-likelihood scores of a match to SOX targeting motif. (DOCX)

S5 Table Primers used for cloning and 5’ RACE analysis. (DOCX)

S1 Files Compressed archive of scripts required for Pydegradome analysis. “Readme.txt” file with instructions on how to run the analysis, as well as the two scripts required for the analysis are included in the archive.
(ZIP)

S2 Files Compressed archive of scripts used for analyses in the paper. “Readme.txt” file with instructions on how to run the analyses, and several scripts used to analyze the data.
(ZIP)
