# Supplementary material for: Transcriptome-Wide Cleavage Site Mapping on Cellular mRNAs Reveals Features Underlying Sequence-Specific Cleavage by the Viral Ribonuclease SOX
Source: PLoS Pathog. 2015 Dec 8;11(12):e1005305. doi: 10.1371/journal.ppat.1005305 (PMC4672902; doi:10.1371/journal.ppat.1005305)
Supplement: S1 Table — % mapping (no restrictions) indicates the percentage of reads that map to the human genome if the requirement for unique mapping to a previously annotated region of the genome is removed. (DOCX) [file ppat.1005305.s008.docx]

**S1 Table: Number of reads obtained from PARE**

| sample | Total # reads | Reads that passed filtering | % of total | Reads with 19-22 nt tag | % of filtered | Uniquely mapped reads | % | % mapping  (no restrictions) |
| --- | --- | --- | --- | --- | --- | --- | --- | --- |
| GFP – repeat 1 | 47,149,821 | 44,225,375 | 93.8 | 43,954,317 | 99.0 | 12,154,219 | 27.8% | 87.3% |
| SOX – repeat 1 | 49,926,649 | 46,824,385 | 93.8 | 46,587,912 | 99.0 | 8,313,094 | 17.9% | 83.5% |
| GFP – repeat 2 | 41,144,429 | 38,188,744 | 92.8 | 37,984,094 | 98.8 | 8,587,943 | 22.8% | 86.0% |
| SOX – repeat 2 | 45,762,349 | 42,674,913 | 93.3 | 42,481,786 | 99.0 | 5,930,358 | 14.0% | 81.5% |
