# Supplementary material for: Transcriptome-Wide Cleavage Site Mapping on Cellular mRNAs Reveals Features Underlying Sequence-Specific Cleavage by the Viral Ribonuclease SOX
Source: PLoS Pathog. 2015 Dec 8;11(12):e1005305. doi: 10.1371/journal.ppat.1005305 (PMC4672902; doi:10.1371/journal.ppat.1005305)
Supplement: S2 Table — The number of peaks detected by using each of the samples as test or control in the PyDegradome program (and plotted in Fig 2A) is listed. Parameters used for this analysis were a scanning window of 4 nt, a multiplicative factor of 4, a confidence level of 99.99%. (DOCX) [file ppat.1005305.s009.docx]

**S2 Table: Number of peaks detected**

| test sample | control sample | peaks detected |
| --- | --- | --- |
| SOX repeat 1 | GFP repeat 1 | 1055 |
| SOX repeat 2 | GFP repeat 2 | 1349 |
| GFP repeat 1 | SOX repeat 1 | 718 |
| GFP repeat 2 | SOX repeat 2 | 416 |
| GFP repeat 1 | GFP repeat 2 | 45 |
| GFP repeat 2 | GFP repeat 1 | 23 |
| SOX repeat 1 | SOX repeat 2 | 74 |
| SOX repeat 2 | SOX repeat 1 | 28 |
