# Supplementary material for: Transcriptome-Wide Cleavage Site Mapping on Cellular mRNAs Reveals Features Underlying Sequence-Specific Cleavage by the Viral Ribonuclease SOX
Source: PLoS Pathog. 2015 Dec 8;11(12):e1005305. doi: 10.1371/journal.ppat.1005305 (PMC4672902; doi:10.1371/journal.ppat.1005305)
Supplement: S4 Table — (DOCX) [file ppat.1005305.s011.docx]

**Table S4 – List of human and KSHV with highest log-likelihood scores**

**A) Highest log-likelihood scores for human RNAs.** Fold changes were derived from Clyde and Glaunsinger, 2011; in that study, fold changes were recorded per gene, rather than per transcript. N.d. = not detected.

| **Transcript** | **Log likelihood score (highest in transcript)** | **Fold change in SOX vs. GFP expressing cells** | **Description** |
| --- | --- | --- | --- |
| ENST00000446344 | 11.142 | 0.631 | Uncharacterized protein |
| ENST00000598878 | 10.862 | 0.494 | Methyltransferase-like 6 |
| ENST00000422843/ ENST00000519402/ ENST00000519749 | 10.856 | n.d. | IL2-inducible T-cell kinase |
| ENST00000573910 / ENST00000572992 | 10.780 | 0.585 | RecQ mediated genome instability 2 |
| ENST00000375180/ ENST00000594687 | 10.717 | 0.450 | Family with sequence similarity 120C |
| ENST00000564510 | 10.535 | n.d. | RP11-327F22.4 |
| ENST00000510434/ ENST00000507596/ ENST00000447513/  ENST00000288774 | 10.496 | 0.706 | Peroxisomal biogenesis factor 10 |
| ENST00000592432/  ENST00000397633/  ENST00000465808/  ENST00000397630/  ENST00000591317 | 10.311 | n.d. | Progressive rod-cone degeneration |
| ENST00000471809 | 10.276 | 0.769 | Intraflagellar transport 27 homolog |
| ENST00000541682 | 10.242 | n.d. | Hes family bHLH transcription factor 7 |

**B) Highest log-likelihood scores for KSHV RNAs.**

| **ORF encoded by RNA** | **Log likelihood score (highest in transcript)** | **Description** |
| --- | --- | --- |
| ORF34/ORF35 | 8.171 | Unknown function |
| ORF25/ORF26/ORF27 | 7.942 | Capsid proteins / glycoprotein |
| ORF8/ORF9 | 7.755 | Glycoprotein B / DNA polymerase |
| PAN | 7.616 | Non-coding RNA |
| ORF45/ORF46/ORF47 | 7.587 | Immune regulation and gene regulation / uracil DNA glycosylase / glycoprotein L |
| ORF69 | 7.579 | Nuclear egress |
| ORF21/ORF22 | 7.292 | Thymidine kinase / glycoprotein H |
| ORF65/ORF67/ORF67.5 | 7.222 | Viral capsid / nuclear egress |
| ORF75 | 7.073 | FGARAT |
| ORF50/K8/K8.1 | 7.056 | Lytic cycle induction / transcription / glycoprotein |
