# Supplementary material for: Transcriptome-Wide Cleavage Site Mapping on Cellular mRNAs Reveals Features Underlying Sequence-Specific Cleavage by the Viral Ribonuclease SOX
Source: PLoS Pathog. 2015 Dec 8;11(12):e1005305. doi: 10.1371/journal.ppat.1005305 (PMC4672902; doi:10.1371/journal.ppat.1005305)
Supplement: S5 Table — (DOCX) [file ppat.1005305.s012.docx]

**S5 Table: Primers used for cloning and 5’ RACE analysis**

| Primer function | Primer sequence |
| --- | --- |
| PGAM1 RACE inner R | TCAGAGGTCAAGGAGCTGATCC |
| PGAM1 RACE outer R | GGAAGGGATTCTAATACACACACC |
| LIMD1 RACE inner R | CTGAGGTGGGAGGATCACTTGAGC |
| LIMD1 RACE outer R | CAAAAATTAGCCAGGCGTAGCCAG |
| RRAGA RACE outer R | GAAGATATTGTCTCGCTGGCTGGTG |
| MAPK8IP3 RACE outer R | GGCTGCAGCCCTCGGTGGCCAGC |
| SRSF3 RACE outer R | ACGAACAGCCATGTATCCATATACAGC |
| BLOC1S4 RACE outer R | GGATTGAATAACAAGGCTTTAATGGCTC |
| CDC5A RACE outer R | GCAGGGCAGCCTTCAAATCCACAC |
| LIMD1 200 nt insertion F | GGCTGTACACCCATGGGCTTCTGTCACACA |
| LIMD1 200 nt insertion R | GGCTGTACAGAGTTTTAACAAGCGCATCAAGC |
| LIMD1 100 nt insertion F | GCTGTACATCTTTGCAGTCCTTTCAAGGCTG |
| LIMD1 100 nt insertion R | GCTGTACAAATTCTCACACATGATCTTTGCAAGC |
| LIMD1 54 nt insertion F | AGCTGTACATGCTGTGTCCCATGCATGTG |
| LIMD1 54 nt insertion R | AGCTGTACAGCAAGCGTCCCAGCAGCCATC |
| LIMD1 39 nt insertion F (Quikchange to add missing A to 39ΔA nt) | CACAGCTCTACTTTCACATGCATGGGTGTACAGCTC |
| LIMD1 39 nt insertion R (Quikchange to add missing A to 39ΔA nt) | GAGCTGTACACCCATGCATGTGAAAGTAGAGCTGTG |
| LIMD1 39ΔA nt insertion F | CTCGGCATGGACGAGCTGTACACCCATGCATGTGAAGTAGAG |
| LIMD1 39ΔA nt insertion R | CGGGAAGCCATGGCTAAGCTTCTTCCCAGCAGCCATCACAGCTC |
| LIMD1 33 nt insertion F | CTCGGCATGGACGAGCTGTACAATGCATGTGAAAGTAGAGCTG |
| LIMD1 33 nt insertion R | CGGGAAGCCATGGCTAAGCTTCTCAGCAGCCATCACAGCTCTAC |
| RRAGA 200 nt insertion F | AGCTGTACACCCGGCGGGTGATGCCAAATACAG |
| RRAGA 200 nt insertion R/ RACE inner R | CGCTGTACAAGTCCCACAGGTTCAGCACCAGG |
| MAPK8IP3 200 nt insertion F | AGCTGTACAGCCCCCAGCCGACTCCAAGCC |
| MAPK8IP3 200 nt insertion R/ RACE inner R | CGCTGTACAGGCTTGTGGGGCAGCTCCCCAAG |
| SRSF3 200 nt insertion F | AGCTGTACAAAATACAGAAACAACTGGCAAAAATTGAAC |
| SRSF3 200 nt insertion R/ RACE inner R | AGCTGTACAAGGCAACCTCTGCCCAGGATGAG |
| SRSF3 54 nt insertion F | CTCGGCATGGACGAGCTGTACACTATAGTTGAACAAGCAGTCTTTA |
| SRSF3 54 nt insertion R | CGGGAAGCCATGGCTAAGCTTCTCCCTGATGGCCTGTGTTTCAC |
| SRSF3 33 nt insertion F | CTCGGCATGGACGAGCTGTACATTGAACAAGCAGTCTTTAAAAACTG |
| SRSF3 33 nt insertion R | CGGGAAGCCATGGCTAAGCTTCTTTTCACAGCAGTTTTTAAAGACTGC |
| BLOC1S4 200 nt insertion F | AGCTGTACATGGGCTCAAGTGATCCTTCCAC |
| BLOC1S4 200 nt insertion R/ RACE inner R | CGCTGTACAGAAAAGGTAACGCCATTTAGTAAAATCC |
| CDCA5 200 nt insertion F | AGCTGTACAATAAGTTACACCGAGTCTACTTGGCCC |
| CDCA5 200 nt insertion R / RACE inner R | CGCTGTACAGATCATGAGATGCATCCAGGCAG |
| PGAM1 200 nt insertion F | CCCTGTACAAGAAGCAACAGAGCCTGTCTGTC |
| PGAM1 200 nt insertion R | CCCTGTACACACATAACGAAATTCAGGATTGATCCC |
| PGAM1 100 nt insertion F | GCTGTACATCACTGCCTACTGCCTGGGGG |
| PGAM1 100 nt insertion R | GCTGTACACCAGATCCTCTGGGGTCAGGG |
| PGAM1 54 nt insertion F | CTCGGCATGGACGAGCTGTACACTAGTCATTCCAGTGGAAGACG |
| PGAM1 54 nt insertion R | CGGGAAGCCATGGCTAAGCTTCTAACAGTTGTCACATCACCACGC |
| PGAM1 33 nt insertion F | CTCGGCATGGACGAGCTGTACATCCAGTGGAAGACGAATGTAAC |
| PGAM1 33 nt insertion R | CGGGAAGCCATGGCTAAGCTTCTTCACCACGCAGGTTACATTC |
| LIMD1 AAA🡪 CCC Quikchange F | GCAGCCATCACAGCTCTACGGGCACATGCATGGGACACAGC |
| LIMD1 AAA🡪 CCC Quikchange R | GCTGTGTCCCATGCATGTGCCCGTAGAGCTGTGATGGCTGC |
| LIMD1 AAA🡪 TTT Quikchange F | GCTGTGTCCCATGCATGTGTTTGTAGAGCTGTGATGGCTGC |
| LIMD1 AAA🡪 TTT Quikchange R | GCAGCCATCACAGCTCTACAAACACATGCATGGGACACAGC |
| LIMD1 AAA🡪 GGG Quikchange F | GCTGTGTCCCATGCATGTGGGGGTAGAGCTGTGATGGCTGC |
| LIMD1 AAA🡪 GGG Quikchange R | GCAGCCATCACAGCTCTACCCCCACATGCATGGGACACAGC |
| LIMD1 cut site A 🡪 G Quikchange F | CCAGCAGCCATCACAGCCCTACTTTCACATGCATG |
| LIMD1 cut site A 🡪 G Quikchange R | CATGCATGTGAAAGTAGGGCTGTGATGGCTGCTGG |
| SRSF3 AA🡪 CC Quikchange F | CTTTTCACAGCAGTTTGGAAAGACTGCTTGTTCAATGTACAGCTCGT |
| SRSF3 AA🡪 CC Quikchange R | ACGAGCTGTACATTGAACAAGCAGTCTTTCCAAACTGCTGTGAAAAG |
| SRSF3 cut site A 🡪 G Quikchange F | GCTTCTTTTCACAGCAGCTTTTAAAGACTGCTTGTTCAATGTACA |
| SRSF3 cut site A 🡪 G Quikchange R | TGTACATTGAACAAGCAGTCTTTAAAAGCTGCTGTGAAAAGAAGC |
| PGAM1 AA🡪 CC Quikchange F | CATCACCACGCAGGTTACAGGCGTCTTCCACTGGAATGAC |
| PGAM1 AA🡪 CC Quikchange R | GTCATTCCAGTGGAAGACGCCTGTAACCTGCGTGGTGATG |
| PGAM1 cut site A 🡪 G Quikchange F | CACCACGCAGGCTACATTCGTCTTCCACTGGA |
| PGAM1 cut site A 🡪 G Quikchange R | TCCAGTGGAAGACGAATGTAGCCTGCGTGGTG |
| GFP construct (SV40) 3’ UTR probe F (for Northern blot) | CTAGATCATAATCAGCCATAC |
| GFP construct (SV40) 3’ UTR probe R (for Northern blot) | TGTAACCATTATAAGCTGCAA |
| GFP 3’ RACE outer R | CCTCCTTGAAGTCGATGCCCTTC |
| GFP 3’ RACE inner R | CTCGATGCGGTTCACCAGGGTGTC |
| GFP TGAAGT 🡪 TGAGTG F | GCCACCTACGGCAAGCTGACCCTGAGTGTCATCTGCACCAC |
| GFP TGAAGT 🡪 TGAGTG R | GTGGTGCAGATGACACTCAGGGTCAGCTTGCCGTAGGTGGC |
